# Encapsulation Process and Dynamic Characterization of SiC Half-Bridge Power Module: Electro-Thermal Co-Design and Experimental Validation

**DOI:** 10.3390/mi16070824

**Published:** 2025-07-19

**Authors:** Kaida Cai, Jing Xiao, Xingwei Su, Qiuhui Tang, Huayuan Deng

**Affiliations:** School of Mechanical and Electrical Engineering, Guilin University of Electronic Technology, Guilin 541004, China; caikaida@mails.guet.edu.cn (K.C.);

**Keywords:** silicon carbide (SiC), half-bridge power module, double-pulse test, COMSOL thermal simulation, packaging process, micro/nano-manufacturing, dynamic characteristics

## Abstract

Silicon carbide (SiC) half-bridge power modules are widely utilized in new energy power generation, electric vehicles, and industrial power supplies. To address the research gap in collaborative validation between electro-thermal coupling models and process reliability, this paper proposes a closed-loop methodology of “design-simulation-process-validation”. This approach integrates in-depth electro-thermal simulation (LTspice XVII/COMSOL Multiphysics 6.3) with micro/nano-packaging processes (sintering/bonding). Firstly, a multifunctional double-pulse test board was designed for the dynamic characterization of SiC devices. LTspice simulations revealed the switching characteristics under an 800 V operating condition. Subsequently, a thermal simulation model was constructed in COMSOL to quantify the module junction temperature gradient (25 °C → 80 °C). Key process parameters affecting reliability were then quantified, including conductive adhesive sintering (S820-F680, 39.3 W/m·K), high-temperature baking at 175 °C, and aluminum wire bonding (15 mil wire diameter and 500 mW ultrasonic power/500 g bonding force). Finally, a double-pulse dynamic test platform was established to capture switching transient characteristics. Experimental results demonstrated the following: (1) The packaged module successfully passed the 800 V high-voltage validation. Measured drain current (4.62 A) exhibited an error of <0.65% compared to the simulated value (4.65 A). (2) The simulated junction temperature (80 °C) was significantly below the safety threshold (175 °C). (3) Microscopic examination using a Leica IVesta 3 microscope (55× magnification) confirmed the absence of voids at the sintering and bonding interfaces. (4) Frequency-dependent dynamic characterization revealed a 6 nH parasitic inductance via Ansys Q3D 2025 R1 simulation, with experimental validation at 8.3 nH through double-pulse testing. Thermal evaluations up to 200 kHz indicated 109 °C peak temperature (below 175 °C datasheet limit) and low switching losses. This work provides a critical process benchmark for the micro/nano-manufacturing of high-density SiC modules.

## 1. Introduction

SiC power modules, leveraging the physical properties of wide-bandgap semiconductors, have achieved revolutionary improvements over traditional silicon-based IGBT power modules in terms of efficiency, power density, switching frequency, and high-temperature performance. Although cost remains a challenge for widespread adoption, SiC modules have become the preferred technology and are rapidly replacing IGBTs in critical applications demanding high performance, high efficiency, and compact designs, such as electric vehicles, renewable energy generation, high-efficiency power supplies, and fast-charging systems. With the maturation of SiC manufacturing technology, increasing production capacity, and continuous cost reduction, the application scope of SiC modules will further expand. This is particularly true for applications requiring high efficiency, high power density, high-temperature operation, and high-frequency switching. Compared to IGBT power modules, the main advantages of SiC power modules include the following [1]:

1. Higher switching frequencies: SiC modules can operate at frequencies significantly higher than IGBTs (typically <20 kHz for optimal IGBT performance vs. up to 100 kHz or MHz range for SiC). This enables the use of smaller, lighter passive components (inductors, capacitors, and transformers), substantially reducing system volume/weight and increasing power density [2].
2. Lower switching losses: SiC exhibits switching losses approximately one order of magnitude lower than comparable IGBTs [3]. At identical switching frequencies, this translates to significantly higher system efficiency. Reduced switching losses also lower cooling requirements, allowing for smaller, lighter heat sinks and reduced thermal management costs.
3. Superior high-temperature performance: SiC devices support significantly higher maximum junction temperatures (Tjs) than silicon devices (typically 175–200 °C+ for SiC vs. 150–175 °C for IGBTs). SiC modules can reliably operate at higher ambient temperatures, deliver increased power output under equivalent cooling conditions, exhibit superior high-temperature stability, and significantly reduce thermal management complexity and cost [4].
4. Higher system efficiency and power density: SiC modules enable power conversion systems with higher overall efficiency, smaller size, reduced weight, and greater power density. This is critical for applications such as electric vehicles, renewable energy (PV inverters and wind converters), data center power supplies, industrial motor drives, and charging stations [5].

In summary, compared to traditional silicon-based IGBTs, SiC technology enables system switching frequencies exceeding 100 kHz, reduces conduction losses by approximately 40%, and maintains stable operation at junction temperatures up to 175 °C [6]. This significantly enhances energy conversion efficiency while reducing system volume. Furthermore, SiC devices outperform their silicon counterparts in high-voltage/high-current operating conditions [7]. Nevertheless, the following challenges persist in optimizing switching characteristics and thermal management design:

1. Dynamic switching stress: The high switching speed of SiC devices induces significant voltage overshoot and electromagnetic interference (EMI) [6]. The switching transient process exhibits extremely high di/dt (current slew rate) and dv/dt (voltage slew rate), causing turn-off voltage spikes exceeding 30% of the bus voltage. This not only exacerbates EMI but also risks gate oscillations and potential device breakdown.
2. Thermal management bottleneck: Under high power density operation, non-uniform thermal distribution within the module compromises system reliability. Current research predominantly focuses on static parameter testing of discrete SiC devices (e.g., using Rogowski coils for current sampling), leaving a significant gap in the co-analysis of static and dynamic behavior for half-bridge power modules. Therefore, this study employs double-pulse testing combined with multiphysics simulation to conduct an in-depth investigation into the switching characteristics and thermal behavior of SiC half-bridge power modules.

Current design analyses of SiC power modules predominantly focus on either standalone electro-thermal coupling simulations or independent process reliability assessments, lacking effective integration for comprehensive synergistic analysis.

Table 1 summarizes the current research landscape for representative SiC half-bridge power modules, both domestically and internationally. Key findings from recent studies include the following:

In a research paper [8], Ze N. and Om P.Y. et al. established electro-thermal models for SiC inverter modules to extract temperature distribution profiles, providing a theoretical thermal reference for power cycling tests.

In the work of [9], the study performed coupled electro-thermal simulation analysis on SiC half-bridge power modules, revealing aging phenomena induced by SiC temperature rise.

In study [10], Yang Y.Y. and Ge Y.X. et al. developed a multiphysics co-simulation methodology (PSpice-MATLAB-COMSOL), laying a theoretical foundation for the electro-thermal design of SiC power modules.

According to review [11], He S.W. and Que L.C. et al. conducted specific design and experimental validation of practical SiC module packaging processes (e.g., substrate material selection, wire bonding), demonstrating the reliability of the implemented packaging design.

In the work of [12], Zhang Z.C. and Justin L. et al. optimized wire bonding techniques by implementing lead-free bonding interconnects. Experimental results validated that this approach reduced parasitic inductance in the package interconnections.

Addressing the current lack of in-depth synergistic analysis between electro-thermal coupling models and process reliability, this study establishes a closed-loop methodology of “design-simulation-process-validation”. This framework is applied to validate the packaging process and dynamic characteristics of a SiC half-bridge power module. Section 2 details the hardware and software design of the double-pulse test board for the SiC half-bridge module, including the following: (1) hardware design of the double-pulse test board; (2) software programming for the double-pulse test board; (3) LTspice simulation analysis of the double-pulse test circuit [13,14]. Section 3 presents the COMSOL steady-state thermal simulation analysis, covering the following: (1) construction of the COMSOL thermal simulation model; (2) configuration of thermal boundary conditions; (3) analysis of multiphysics thermal simulation results. Section 4 elaborates on the packaging process flow for the SiC half-bridge module, encompassing the following: (1) conductive adhesive sintering experiments for SiC MOSFET wafers; (2) high-temperature baking experiments; (3) aluminum wire bonding experiments. Section 5 describes the dynamic characteristic testing and analysis of the SiC half-bridge module, featuring the following: (1) introduction of the experimental test platform; (2) double-pulse test waveform analysis; (3) double-pulse test timing analysis; (4) analysis of drain–source voltage ($U_{ds}$) waveforms for the lower MOSFET; (5) Analysis of drain current ($I_{D}$) waveforms for the lower MOSFET. Finally, Section 6 discusses the potential value and application prospects of the developed SiC half-bridge power module.

## 2. Hardware and Software Design of Double-Pulse Test Board for SiC Half-Bridge Power Module

The double-pulse test board for SiC half-bridge power modules is equipped with short-circuit protection and under-voltage protection. It supports wide-range voltage input and multi-channel voltage output, and the driving negative voltage range is adjustable, which can be used to compatibly drive both MOS and IGBT.

### 2.1. Hardware Design of Double-Pulse Test Board

**Figure 1:**
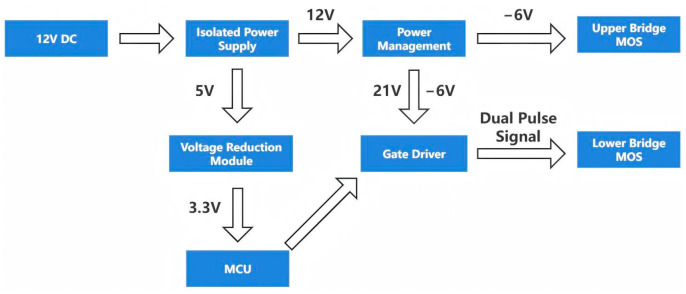
Hardware system block diagram of double-pulse test board.

Figure 1 presents the hardware system block diagram of the double-pulse test board, with the STM32 microcontroller made by STMicroelectronics in Geneva, Switzerland the MCU. A 12 V DC voltage is input to the isolated power module F2405S-2WR3 developed by Jinke Electronics Manufacturer in Shenzhen, China, which outputs 12 V and 5 V. The 5 V voltage is stepped down to 3.3 V by the AMS1117 voltage-reduction module made by Advanced Monolithic Systems Inc. in Livermore, CA, USA to power the MCU, while the 5 V voltage is also supplied to the gate driver IC IVCO1A01DWR developed by Inventchip Manufacturer in Shanghai, China [15]. A 12 V voltage is fed into the power management chip VPS8703B made by Yuante Semiconductor Manufacturer in Suzhou, China; after voltage-doubling rectification, two isolated power supplies of 21 V and −6 V are generated. One −6 V supply acts as the gate voltage for the upper-bridge MOS to ensure it remains turned off continuously. The two isolated power supplies (21 V and −6 V) are fed into the gate driver IC IVCO1A01DWR. When the gate driver IC receives the trigger pulse signal from STM32, it outputs a 21 V driving voltage to the lower-bridge MOS; when the gate driver IC does not receive the trigger pulse signal from the STM32, it outputs a −6 V driving voltage to the lower-bridge MOS. The OLED screen communicates with the MCU via the I2C protocol, and the PA8 pin of STM32 is utilized as the PWM trigger pin for the lower bridge.

**Figure 2:**
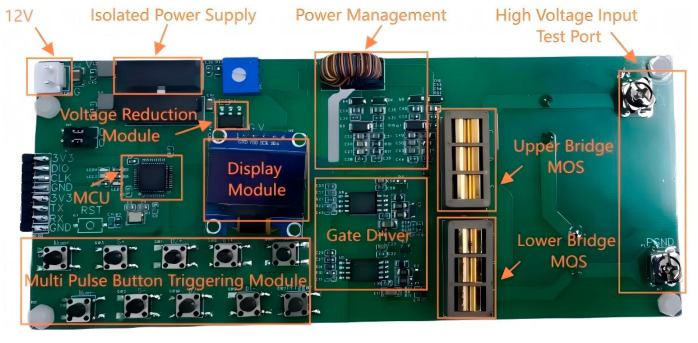
Hardware composition of the double-pulse test board.

Figure 2 shows the hardware composition of the double-pulse test board, which is primarily composed of an isolation power supply module, an MCU module, a display module, a multi-pulse button triggering module, a power management module, and a gate driver module.

### 2.2. Software Program Design of Double-Pulse Test Board

**Figure 3:**
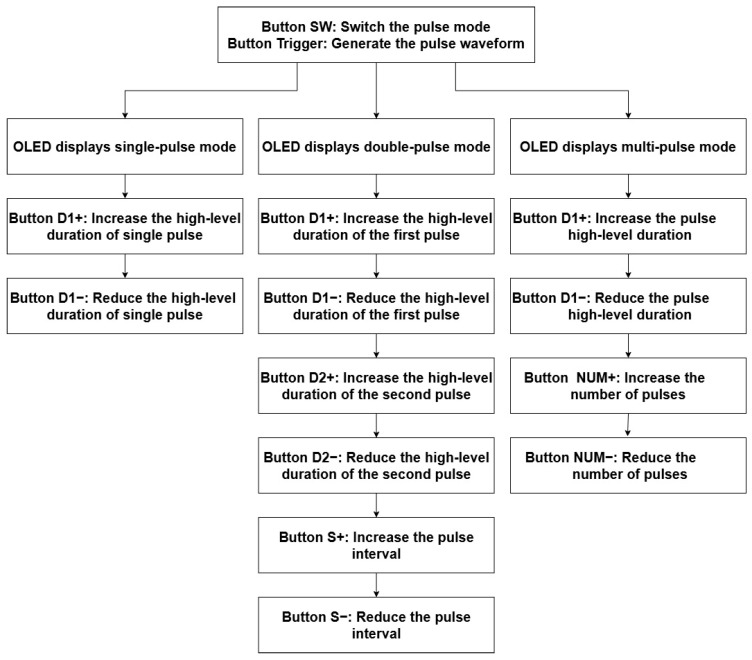
Flowchart of software program design for double-pulse test board.

Figure 3 presents the software program flowchart for the double-pulse test board. This system supports switching between three operating modes: single-pulse, double-pulse, and multi-pulse. In multi-pulse mode, up to 10 pulses can be configured.

Pulse timing parameters (pulse width, interval, and number) are adjustable via the following eight dedicated keys: S+, S−, D1+, D1−, D2+, D2−, NUM+, and NUM−.

The double-pulse mode employed in this study produces waveforms comprising two distinct high-level signal segments separated by an intervening low-level interval.

### 2.3. LTspice Simulation Analysis of Double-Pulse Test Circuit

**Figure 4:**
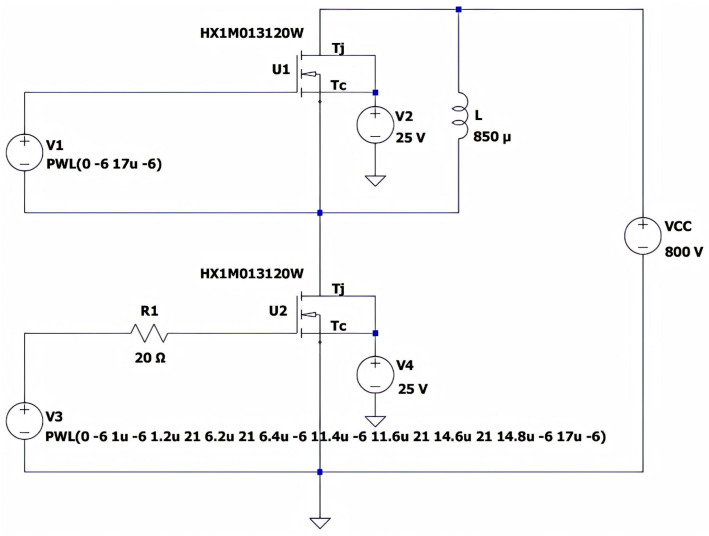
LTspice simulation design diagram of double-pulse test board.

Figure 4 presents the LTspice simulation schematic of the double-pulse test board. During simulation, the upper-switching-arm SiC MOSFET in the half-bridge power module was biased at a negative voltage to maintain the OFF-state [13,16]. The lower-switching-arm MOSFET functioned as the device under test (DUT), requiring measurement of the drain–source voltage ($V_{ds}$), drain current ($I_{d}$), and gate–source voltage ($V_{gs}$).

Table 2 presents the LTspice simulation parameters for the double-pulse test circuit of the SiC half-bridge power module [17]. The HX1M013120W SiC MOSFETs from Macrocore Semiconductor (Shenzhen, China) were used in both the upper and lower bridge arms.

**Figure 5:**
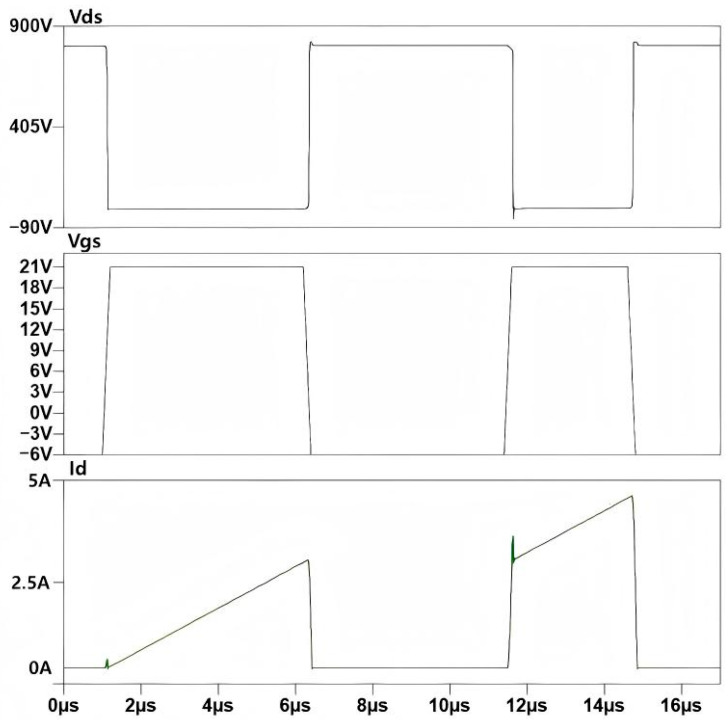
LTspice simulation waveform diagram of double-pulse test board.

Using LTspice, double-pulse gate-drive signals were applied to the lower-bridge-arm MOSFET in the simulation shown in Figure 4. The resulting current and voltage switching characteristics are illustrated in Figure 5, which can be divided into the following three stages:

1. The first pulse duration $T_{1}$

Upon triggering the first pulse, the gate–source voltage $V_{gs}$ of the DUT rises to the turn-on driving voltage $V_{on}$, enabling DUT conduction. Its drain–source voltage $V_{ds}$ drops from the bus voltage VCC to the on-state voltage [18,19]. Energy stored in the bus capacitor is transferred to the load inductor L via the activated DUT [20,21]. The bus voltage is almost entirely applied across the load inductor. Assuming the bus capacitor is sufficiently large (i.e., bus voltage remains constant during switching), the DUT’s drain current increases linearly to the specified current $I_{m}$, which can be expressed as follows:

(1) $$I_{m}=\frac{V_{CC}T_{1}}{L}$$

where the first pulse duration $T_{1}$ is set as 5 μs. From Table 2, $V_{CC}=800\;V$ and L=850 μH. Thus, the specified current $I_{m}$ calculated via Equation (1) is 4.71 A. With VCC (bus voltage) and L (load inductance) fixed, the value of the specified test current $I_{m}$ can be independently adjusted by controlling the pulse width of $T_{1}$.

2. The pulse interval $T_{2}$

After the first pulse ends, the DUT’s gate–source voltage $V_{gs}$ drops to the turn-off voltage $V_{off}$, turning off the DUT [22,23]. Current then freewheels through the body diode of the upper-bridge MOSFET. With parasitic inductance, the drain–source voltage $V_{ds}$ exhibits additional spikes during turn-off process. The pulse interval $T_{2}$ should not be shorter than the time period from the start of $V_{gs}$ oscillation to its stabilization. Moreover, an excessively long pulse duration will also cause a notable temperature rise in the device. In this study, $T_{2}$ is set to 5 μs.

3. The second pulse duration $T_{3}$

When the DUT is turned on again, the current transitions from the freewheeling loop to the DUT. At this stage, the current flowing through the DUT is the superposition of the inductor current and the reverse recovery current of the body diode [24,25]. The current of the DUT continues to rise linearly until the second pulse ends.

The duration of the second pulse $T_{3}$ should not be excessively long to prevent the current from exceeding the device’s safe operating threshold. Additionally, an overly high drain current $I_{d}$ will cause excessive turn-off voltage overshoot, which may exceed the device’s voltage rating and damage the device. For discrete devices, $T_{3}$ generally does not exceed 10 μs. For power modules, it typically does not exceed 50 μs. For the SiC half-bridge power module designed in this study, $T_{3}$ is set to 3 μs.

Figure 5 presents the $V_{gs}$, $V_{ds}$, and $I_{d}$ waveforms of the DUT under the simulation setup specified in Table 2. As observed from Figure 5, the drain current $I_{D}$ flowing through the lower-bridge MOSFET is approximately 4.65 A, which is generally consistent with the theoretical value of 4.71 A calculated via Equation (1). The simulated value is slightly lower than the theoretical value, and this discrepancy is attributed to power losses in the freewheeling loop when the gate–source voltage $V_{gs}$ of the DUT drops to the turn-off voltage $V_{off}$ after the first pulse ends, with current freewheeling through the body diode of the upper-bridge MOSFET [22]. The gate-drive voltage $V_{gs}$ amplitude for the lower bridge is 21 V, while the drain–source voltage $V_{ds}$ amplitude is 800 V.

As noted in article [26], limited probe bandwidth and current–voltage probe delay mismatch prevent the attainment of the desired measurement accuracy. To alleviate this situation, a high-bandwidth probe (100 MHz) with the smallest grounding loop was used, and probe calibration was performed under no-load conditions.

### 2.4. Q3D-Based Extraction of Parasitic Inductance in SiC Half-Bridge Modules

**Figure 6:**
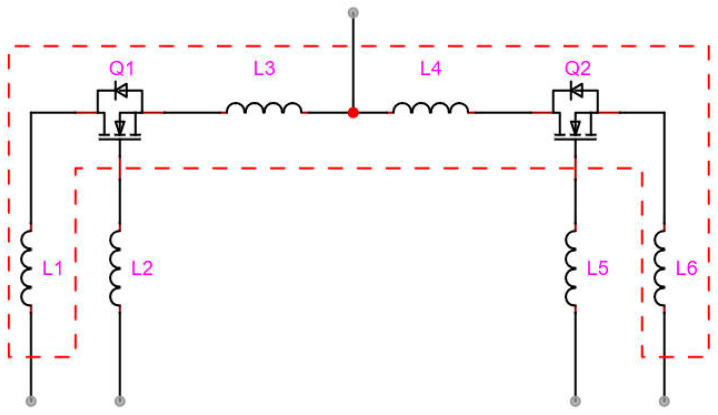
Parasitic inductance extraction scheme using Ansys Q3.

The extremely fast switching speed of SiC MOSFET chips significantly amplifies the effects of parasitic parameters, necessitating minimization of these parameters in the loop. To accurately analyze parasitic parameters within the module, Ansys Q3D 2025 R1 finite element analysis software was employed. The specific methodology involved importing the 3D model of the module into Q3D and setting the frequency to 25 kHz, with the parasitic parameter distribution extracted from the simulation results shown in Figure 6.

Parasitic parameters in the module reside in the power loop and driver loop. The parasitic parameters of the power loop are delineated by the dashed red box in Figure 6. Table 3 defines the internal parasitic inductances of the module and their impacts on switching characteristics, where the power loop parasitic inductance is identified as the dominant factor causing voltage overshoot and oscillation during switching transitions. Due to the extremely fast switching speed of SiC MOSFETs, their performance exhibits exceptional sensitivity to parasitic inductance. Consequently, minimizing parasitic inductance in the power loop constitutes one of the primary design objectives for the module. As indicated in Table 3, the parasitic inductance within the dashed red box of the power loop in Figure 6 is 6 nH.

## 3. COMSOL Steady-State Thermal Simulation Analysis of SiC Half-Bridge Power Module

### 3.1. Construction of COMSOL Thermal Simulation Model

The SiC half-bridge power module investigated in this study is composed of two SiC wafers. The 3D model constructed using SOLIDWORKS was imported into COMSOL, and the main material structure dimensions and central coordinates are presented in Table 4.

**Figure 7:**
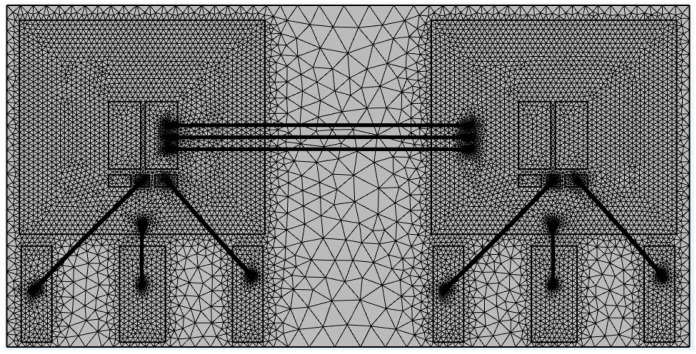
Mesh generation of SiC half-bridge power module.

Considering computational complexity and model accuracy, refined mesh division was performed on bond wires, solder layers, and die layers. The mesh comprises 298,223 elements, and the mesh division of the SiC half-bridge power module is illustrated in Figure 7.

In the COMSOL simulation setup for the silicon carbide half-bridge power module, the SiC MOSFET wafer material was defined as SiC, the substrate material as copper, and the bond wire material as aluminum. Primary material parameters included thermal conductivity, density, and heat capacity. Table 5 shows the information on the model of MOSFETs used in computations.

Table 6 details the list of parameters values of the used model. The MOSFET model HX1M013120W was selected, with its designation identical to that in the LTspice simulation schematic of the double-pulse test board shown in Figure 4.

### 3.2. Thermal Field Boundary Condition Setting

**Figure 8:**
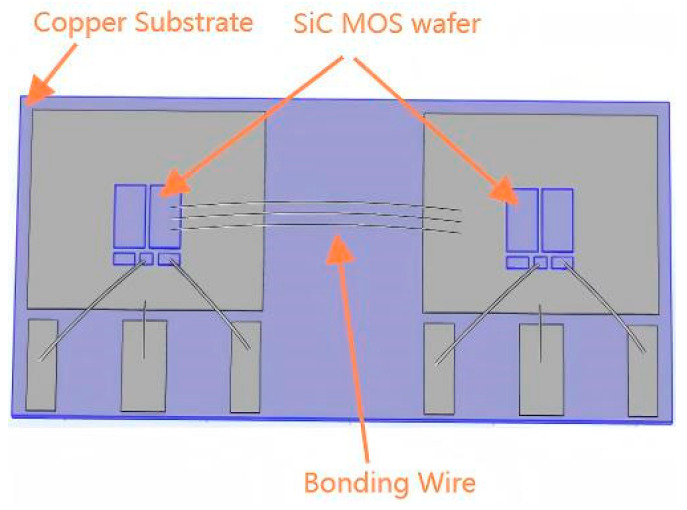
Packaging structure of SiC half-bridge power module.

Figure 8 illustrates the packaging structure of the SiC half-bridge power module, whose core components primarily include a copper baseplate, two SiC wafers, and bond wires. The copper baseplate serves as the main heat dissipation path, while the power losses of the two SiC MOS wafers act as the primary heat sources.

In the thermal simulation model of the SiC half-bridge power module established in this study, simplified thermal boundary conditions were defined based on the module’s physical structure and actual operating conditions to simulate its temperature distribution characteristics in real-world operating environments.

Regarding the definition of thermal boundary conditions, full consideration was given to the actual operating scenarios of the SiC half-bridge power module. In practical applications, the bottom surface of the copper baseplate in the MOS module is typically tightly attached to the heat sink via thermal grease, and this attachment method significantly affects the module’s heat dissipation efficiency. To accurately replicate this heat dissipation process, the bottom surface of the copper baseplate was defined as a convective heat flux boundary in the simulation, with a heat transfer coefficient set to 1500 W/(m^2^∙K). This heat transfer coefficient was determined comprehensively based on multiple factors, including the thermal conductivity of the thermal grease and the contact status between the heat sink and the copper baseplate, enabling a relatively accurate simulation of the heat exchange process between the bottom surface of the copper baseplate and the heat sink [27].

For the other boundaries of the module, considering the thermal convection phenomenon between the module and the surrounding air in the actual operating environment, all boundaries except the bottom surface of the copper baseplate were defined as being in contact with air, with a heat transfer coefficient set to 5  W/(m^2^∙K). This heat transfer coefficient corresponds to the heat exchange capability between air and the module surface under natural convection conditions.

Meanwhile, to further simulate thermal convection in real-world environments, an infinite air domain was constructed around the model, and its temperature was set constant at 25 °C. The establishment of this air domain can avoid distortion in thermal convection simulation caused by boundary limitations, making the simulation results closer to the heat dissipation state of the MOS module under actual operating conditions. Thus, it provides reliable theoretical data support for evaluating the module’s thermal performance and optimizing heat dissipation design.

Through the definition of the above-mentioned series of boundary conditions, a thermal simulation model that not only conforms to actual physical phenomena but also facilitates calculation and analysis was constructed, laying a simulation foundation for studying the thermal characteristics of the SiC half-bridge power module in a real-world environment.

First, the power loss of the SiC MOS wafer must be calculated based on the datasheet and loss formulas. The total power loss $P_{\mathrm{loss}}$ of the MOS mainly consists of conduction loss $P_{\mathrm{con}}$ and switching loss $P_{\mathrm{sw}}$, with the calculation formulas as follows:

(2) $$P_{\mathrm{loss}}=P_{\mathrm{con}}+P_{\mathrm{sw}}$$

(3) $$P_{\mathrm{con}}={I^{2}}_{d\text{\_}\mathrm{rms}}\cdot R_{\mathrm{ds}}\cdot D$$

(4) $$P_{\mathrm{sw}}={(E}_{on}+E_{off})\cdot f_{\mathrm{sw}}$$

where $P_{\mathrm{loss}}$ represents the total power loss, $P_{\mathrm{con}}$ denotes the conduction loss, and $P_{\mathrm{sw}}$ stands for the switching loss. $I_{d\text{\_}\mathrm{rms}}$ is the root mean square (RMS) current during the MOS conduction period, $R_{\mathrm{ds}}$ is the drain–source on-resistance of the MOS, and D is the conduction duty cycle of the MOS [28]. $E_{on}$ is the turn-on energy loss, $E_{off}$ is the turn-off energy loss, and $f_{\mathrm{sw}}$ is the switching frequency.

Table 7 presents the main parameters for power loss calculation (based on the SiC MOS wafer datasheet).

Based on Equations (2)–(4) and the parameters in Table 4, the conduction loss $P_{\mathrm{con}}$ of the SiC MOS wafer is calculated as 115 W, the switching loss $P_{\mathrm{sw}}$ as 2.625 W, and the total power loss $P_{\mathrm{loss}}$ as 117.625 W.

### 3.3. The Analysis of Thermal–Physical Field Simulation Results

**Figure 9:**
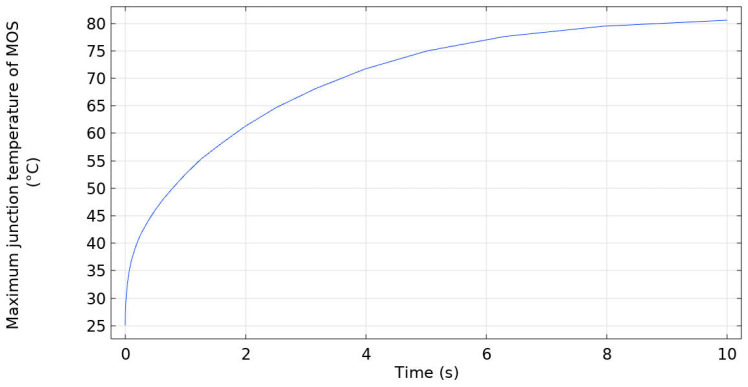
The temperature curve diagram of the maximum junction temperature of the SiC half-bridge power module.

Figure 9 presents the temperature curve of the maximum junction temperature of the SiC half-bridge power module, with the ambient temperature set at 25 °C. As time elapses, the maximum junction temperature of the MOS rises rapidly; at approximately 1 s the temperature exceeds 50 °C and continues to increase thereafter, reaching around 80 °C at about 8 s and then stabilizing.

This phenomenon indicates that, in the initial stage, the junction temperature of the MOS rises rapidly because the wafer—acting as a continuous heat source—generates heat that accumulates inside the module. As time further increases, although the wafer continues to generate heat, heat exchange between the module and the external environment (particularly convective heat dissipation between the bottom surface of the copper baseplate and the heat sink, with a configured heat transfer coefficient of 1500  W/(m^2^∙K)) enables the heat to gradually reach a dynamic equilibrium. Consequently, the temperature no longer rises significantly, and the maximum junction temperature of the MOS eventually stabilizes.

**Figure 10:**
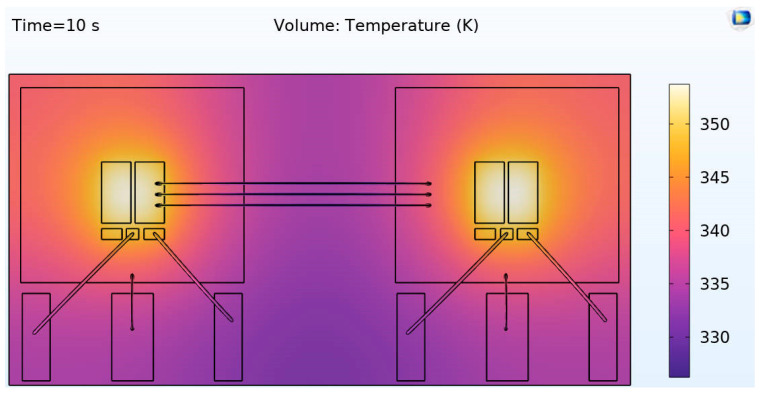
The temperature distribution diagram of the maximum junction temperature of the SiC half-bridge power module.

Figure 10 illustrates the temperature distribution of the maximum junction temperature in the SiC half-bridge power module. COMSOL Multiphysics 6.3 simulations demonstrate that under a power loss of 117.6 W, the junction temperature gradient of the module reaches 55 °C (from 25 °C to 80 °C), with local hotspots predominantly concentrated at the SiC wafers.

The thermal–physical field simulation results reveal that heat sources are predominantly concentrated at the SiC wafers, providing optimization insights for practical thermal management design. For example, adopting heat sinks with superior thermal performance and optimizing the application process of thermal grease at the SiC wafers to enhance thermal conductivity efficiency, thereby ensuring the MOS module operates within a safe temperature range during long-term operation and improving the reliability and stability of the SiC half-bridge power module.

## 4. Packaging Process Preparation Flow of the SiC Half-Bridge Power Module

### 4.1. Conductive Adhesive Sintering Experiment of SiC MOS Wafers

**Figure 11:**
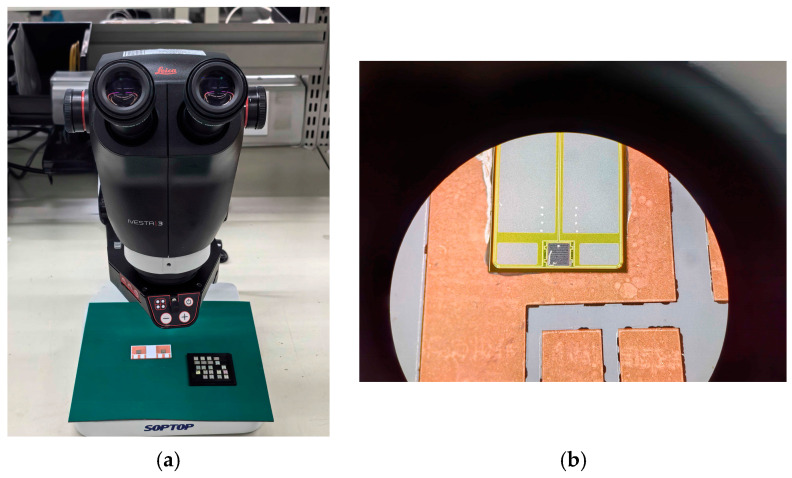
(**a**) Visual defect inspection via microscope. (**b**) Conductive adhesive sintering of SiC MOS wafers onto the copper substrate. Schematic of conductive adhesive sintering for the SiC half-bridge power module.

Figure 11 illustrates the schematic of conductive adhesive sintering for the SiC half- bridge power module. As shown in Figure 11a, visual defect inspection of the copper baseplate and SiC wafers in the SiC half-bridge power module was performed using a Leica IVesta 3 microscope made by Leica Microsystemsstereo Manufacturer in Wetzlar, Germany. The IVesta 3 employs FusionOptics technology, which offers the dual advantages of high resolution and reduced refocusing time. Its FusionOptics technology enables observation in 3D mode, where the focusing area is increased without compromising clarity. Additionally, the time required for microscope adjustment is reduced, allowing for immediate defect identification.

In this experiment, the conductive adhesive S820-F680 developed by Silanex Technology Manufacturer in Taizhou, China was selected, which features high electrical conductivity, solvent resistance, a thermal conductivity of 39.3  W/m·K, and an operating temperature range from −40 °C to 175 °C. It is widely used in certain fields, such as semiconductor die packaging, module packaging, 5G signal devices, and communication modules, where high temperature and voltage resistance are required.

As illustrated in Figure 11b, leveraging the microscope’s maximum magnification of 55× and apochromatic correction capability, defect inspection of the sintering/bonding interface was conducted to assess the actual sintering/bonding performance.

### 4.2. High-Temperature Baking Experiment

**Figure 12:**
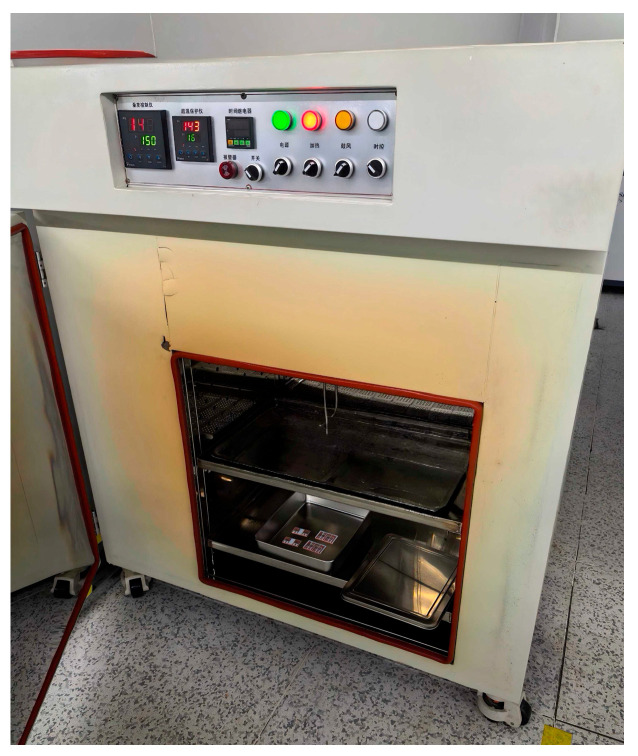
Schematic of high-temperature baking for SiC half-bridge power module.

Figure 12 illustrates the schematic of high-temperature baking for the SiC half-bridge power module. The copper baseplate of the SiC half-bridge power module was placed into a high-temperature oven (model: SH881-5 made by Sanhe Manufacturer in Suzhou, China). This oven operates within a temperature range of 25–300 °C, with a temperature control accuracy of ±1 °C and a heating power of 3.9 kW. Baking was carried out at 150 °C for 2 h. In the high-temperature environment, the SiC MOS wafers and the copper baseplate were bonded together via conductive adhesive. 

### 4.3. Aluminum Wire Bonding Experiment

**Figure 13:**
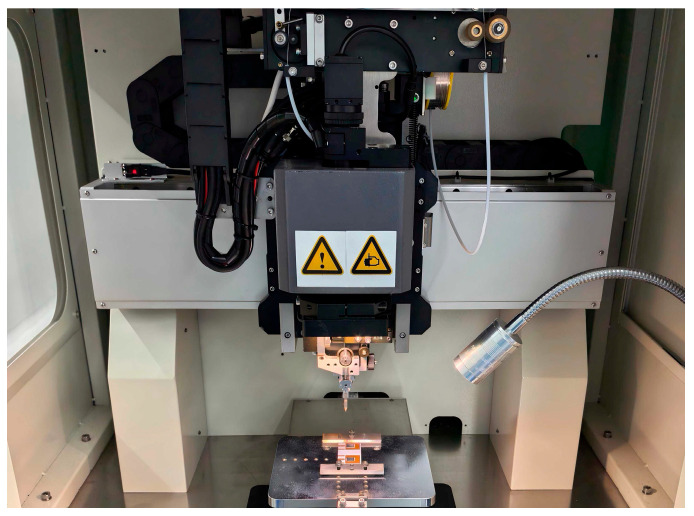
Schematic of aluminum wire bonding for SiC half-bridge power module.

Figure 13 illustrates the schematic of aluminum wire bonding for the SiC half-bridge power module. In the physical fabrication of the SiC half-bridge power module, the wire bonding process is a core step to ensure the module’s electrical performance and reliability. In this experiment, 15 mil aluminum wires were used as bonding wires, and a self-service ultrasonic aluminum wire bonder was employed to perform the wire bonding operation. The 15 mil aluminum wires achieve a good balance in electrical conductivity, mechanical strength, and cost, which can meet the electrical connection requirements of the SiC half-bridge power module. Meanwhile, the self-service ultrasonic aluminum wire bonder enables more precise wire bonding operations.

First, preprocessing was carried out for the SiC half-bridge power module. The wafer surface and the bonding area on the copper baseplate were cleaned to remove surface oil, oxides, and other impurities, so as to prevent these contaminants from impacting the bonding interface [2,29]. After cleaning, the SiC half-bridge power module was installed on the bonder workbench, and a high-precision positioning fixture was utilized to ensure the module is fixed in position.

During the parameter-setting stage, welding parameters were specifically formulated based on the specifications of aluminum wires and the characteristics of bonding materials. The contact point where the aluminum wire contacts the copper baseplate was defined as the first contact point. The welding ultrasonic power was set to 750 mW, the welding pressure to 750 g, and the welding time to 180 ms.

The contact point where the aluminum wire contacts the SiC wafer was defined as the second contact point. For the second bonding site, considering the relatively thin metal layer on the SiC wafer surface, the welding ultrasonic power was adjusted to 500 mW, the welding pressure was reduced to 500 g, and the welding time was shortened to 160 ms to avoid SiC wafer damage caused by excessive energy input. This parameter combination ensures reliable bonding between the aluminum wire and the wafer’s surface metal layer, whilst also minimizing welding energy input to protect the wafer. During welding, ultrasonic vibration and pressure act synergistically to promote interatomic diffusion between the aluminum wire and the SiC wafer surface, forming a stable bonding interface and achieving reliable electrical connection.

After completing each wire-bonding cycle, the bonding points were immediately inspected microscopically using a Leica IVesta 3 stereo microscope to evaluate the shape, size, and interface bonding quality of the bonds. Special attention was paid to detecting defects such as cold solder joints, cracks, and voids at the bonding points to ensure compliance with process-defined quality requirements.

## 5. Dynamic Characteristic Testing and Analysis of SiC Half-Bridge Power Module

### 5.1. Double-Pulse Test Experimental Platform

**Figure 14:**
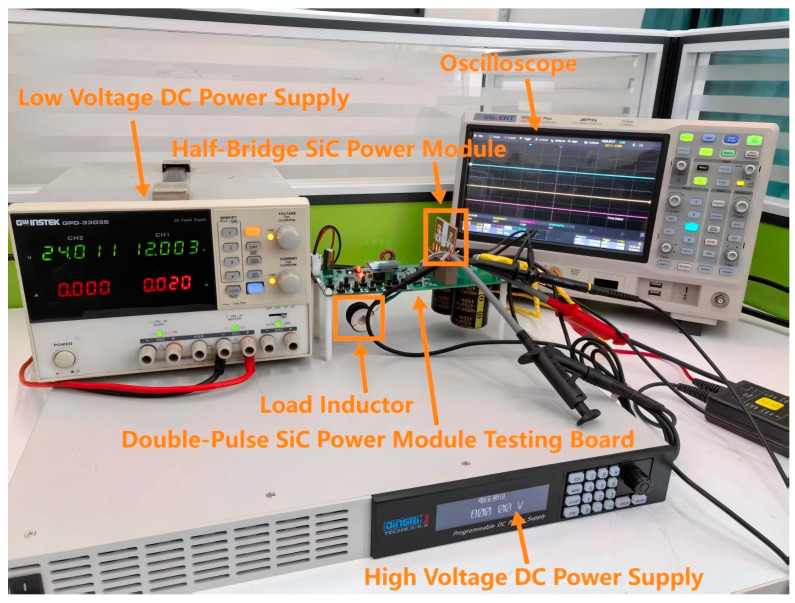
Experimental platform for dynamic characteristic testing of SiC half-bridge power module.

Figure 14 presents the experimental platform for dynamic characteristic testing of the SiC half-bridge power module. A low-voltage DC power supply provides an input voltage of 12 V to the double-pulse test board. A high-voltage DC power supply applies a test voltage of 800 V between the drain and source of the lower-bridge MOS in the SiC half-bridge power module. A high-voltage differential probe is used to measure the drain–source voltage $V_{Ds}$ of the lower-bridge MOS, and a Rogowski coil is used to measure the drain current $I_{D}$ of the lower-bridge MOS [30]. This Rogowski coil has a microsecond-level response accuracy and can accurately capture the dynamic characteristics of the current.

Table 8 presents the main parameters and equipment models of the double-pulse test experimental platform.

### 5.2. Waveform Analysis of Double-Pulse Test

**Figure 15:**
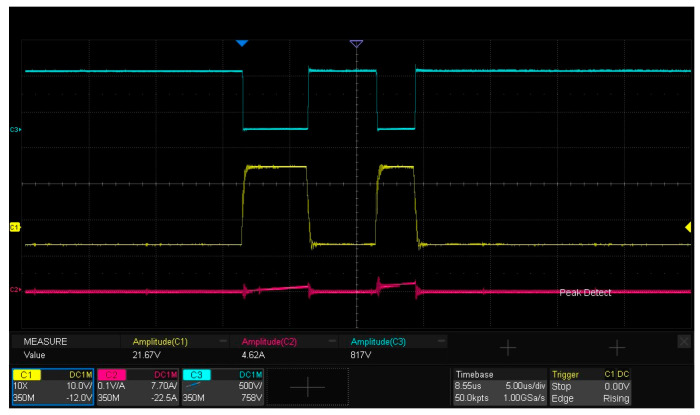
Experimental waveforms of dynamic characteristic testing for the SiC half-bridge power module.

Figure 15 presents the experimental waveforms for dynamic characteristic testing of the SiC half-bridge power module. As observed from the figure, the drain current $I_{D}$ flowing through the lower-bridge MOS is 4.62 A, which is slightly lower than the 4.71 A derived from the calculation of Formula (1). The underlying reason is that after the first pulse ends, when the gate–source voltage $V_{gs}$ of the DUT decreases to the turn-off drive voltage $V_{off}$, the current freewheels through the body diode of the upper MOS, and inherent losses exist in the freewheeling loop. The amplitude of the gate–source drive voltage $V_{gs}$ for the lower bridge is 21.67 V, while the drain–source voltage $V_{ds}$ reaches 817 V. The experimental results are generally consistent with the LTspice simulation analysis results of the double-pulse test circuit in Section 2.3.

### 5.3. Time Analysis of Double-Pulse Test

**Figure 16:**
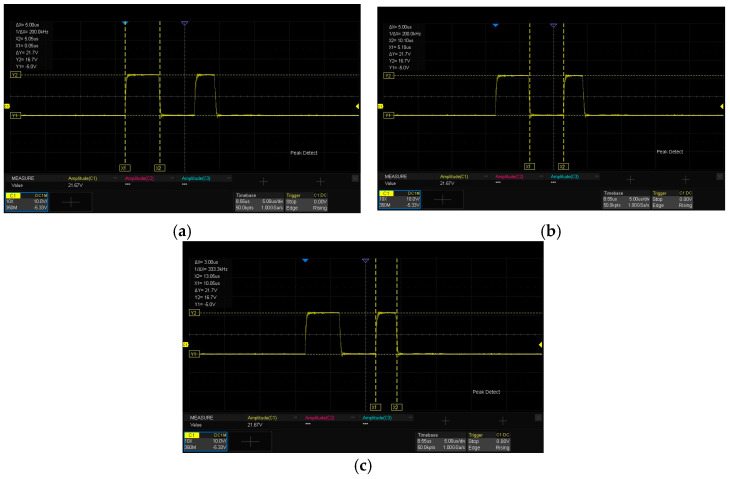
(**a**) The first pulse duration. (**b**) The pulse interval duration. (**c**) The second pulse duration. Waveform of gate drive voltage $V_{gs}$ of the lower-bridge MOS.

Figure 16 illustrates the gate-drive voltage $V_{gs}$ waveform of the lower-bridge MOS in the SiC half-bridge power module. Figure 16a denotes the first pulse duration $T_{1}$, where $T_{1}=5\mu s$. Figure 16b represents the pulse interval duration $T_{2}$, with $T_{2}=5\mu s$. Figure 16c shows the second pulse duration $T_{3}$, and $T_{3}=3\mu s$, which conforms to the time-setting requirements of the LTspice simulation analysis for the double-pulse test circuit in Section 2.3.

### 5.4. Waveform Analysis of Drain–Source Voltage $U_{ds}$ of Lower-Bridge MOS

**Figure 17:**
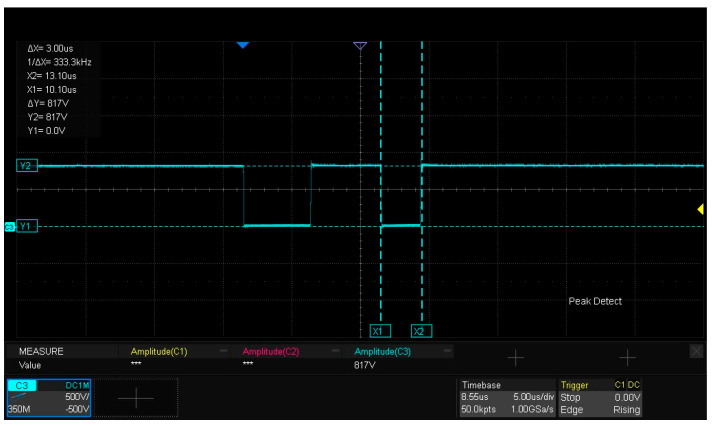
Drain–source voltage $V_{Ds}$ of the lower-bridge MOS.

Figure 17 presents the drain–source voltage $V_{Ds}$ of the lower-bridge MOS in the SiC half-bridge power module. The amplitude of $V_{ds}$ is 817 V, and the experiment verifies that the packaged SiC half-bridge power module exhibits electrical performance characteristics enabling it to withstand a high voltage of 800 V.

### 5.5. Waveform Analysis of Drain Current $I_{D}$ of Lower-Bridge MOS

**Figure 18:**
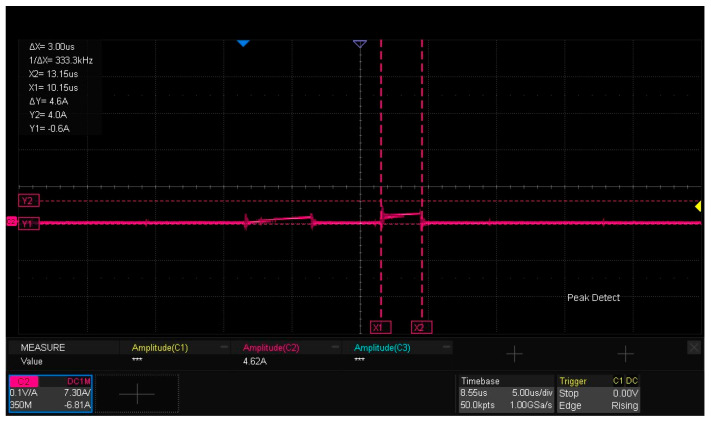
Drain current $I_{D}$ of the lower-bridge MOS.

Figure 18 illustrates the drain current $I_{D}$ of the lower-bridge MOS in the SiC half- bridge power module, with the amplitude of $I_{D}$ being 4.62 A. The experiment verifies the packaged SiC half-bridge power module undergoes double-pulse dynamic performance testing on an 800 V high-voltage experimental platform, and the amplitude of its drain current $I_{D}$ is 4.62 A—slightly lower than the 4.71 A derived from the calculation of Formula (1). The reason is that after the first pulse ends the gate–source voltage $V_{gs}$ of the DUT decreases to the turn-off drive voltage $V_{off}$ and the freewheeling current flows through the body diode of the upper MOS, introducing losses in its path [31,32].

### 5.6. Characterization of Turn-On and Turn-Off Waveforms

**Figure 19:**
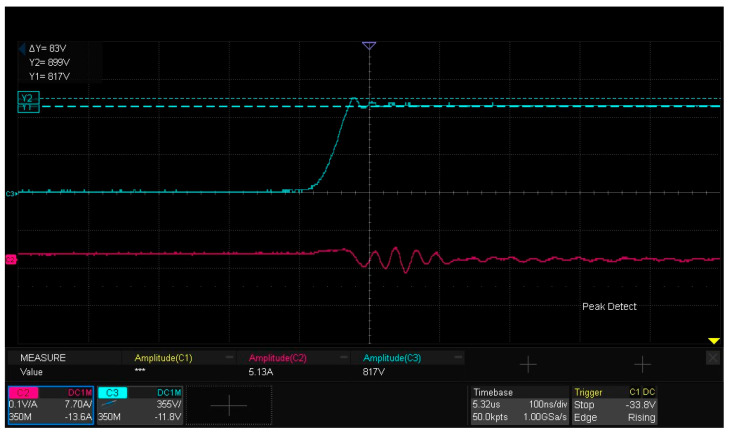
Turn-off waveform: drain–source voltage ($V_{Ds}$) overshoot measurement of lower-arm MOS.

**Figure 20:**
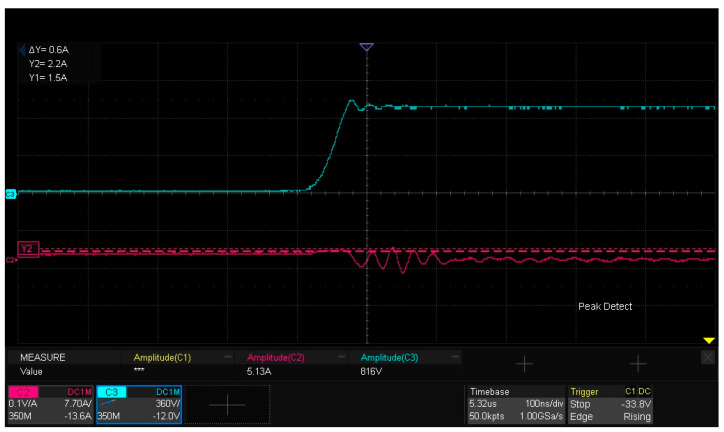
Turn-off waveform: drain current ($I_{D}$) transition measurement of lower-arm MOS.

The turn-on and turn-off waveforms of the double-pulse test are depicted in Figure 18, Figure 19 and Figure 20. Among them, Figure 19 measures the drain–source voltage overshoot of the lower-arm MOSFET during turn-off, revealing an overshoot $\Delta V_{DS}$ of 83 V. Figure 20 captures the drain current $I_{D}$ transient of the lower-arm MOSFET during turn-off, indicating a current change rate di of 0.6 A at a time dt of 0.06 ns. The parasitic inductance $L_{loop}$ of the power loop can be calculated using Formula (5), which utilizes the voltage drop $\Delta V_{DS}$ across the parasitic inductance induced by the current change rate ($\frac{di}{dt}$). The calculated inductance $L_{loop}$ is 8.3 nH and the formula used is as follows:

(5) $$\Delta V_{DS}=L_{loop}\frac{di}{dt}$$

**Figure 21:**
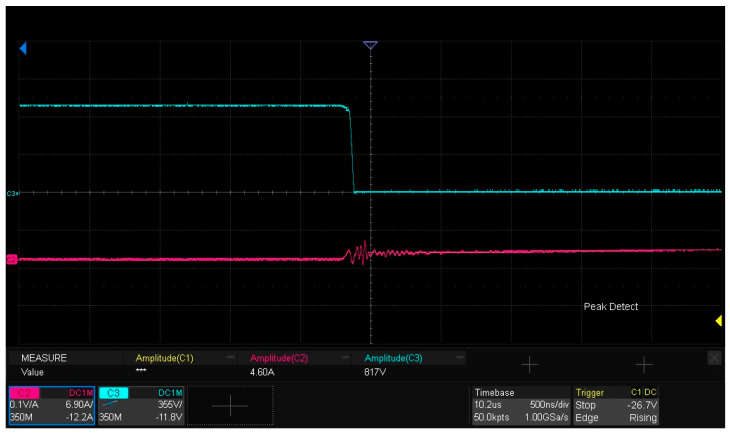
Turn-on waveform: drain–source voltage ($V_{Ds}$) and drain current ($I_{D}$) of lower-arm MOS.

Figure 21 displays the turn-on waveforms of the module. As observed, the drain current $I_{D}$ of the lower-arm MOSFET exhibits damped oscillation after turn-on, while the drain–source voltage $V_{Ds}$ develops minor fluctuations synchronized with the $I_{D}$ oscillation.

The experimental results indicate an intra-module parasitic inductance of 8.3 nH, which is within the acceptable design margin. This value neither induces significant $V_{Ds}$ overshoot nor excessive $I_{D}$ variations. Compared with the simulation result (6 nH), the measured value is marginally higher. This discrepancy may be attributed to additional inductance introduced by the experimental test loop itself, leading to fluctuations of parasitic inductance.

To comprehensively investigate the switching characteristics of the power module—including how frequency impacts losses and thermal performance—systematic double-pulse tests were conducted across a wide operating frequency range (25–200 kHz).

Turn-on loss is the energy dissipated during the device’s transition from the off-state to the on-state, obtained by integrating the instantaneous power over the turn-on transient period. Similarly, turn-off loss is the energy dissipated during the transition from the on-state to the off-state, obtained by integrating the instantaneous power over the turn-off transient period.

The results of the switching characterization are summarized in Table 9, revealing that both turn-on and turn-off losses scale nearly linearly with frequency; for example, turn-on loss jumps from 65 μJ at 25 kHz to 450 μJ at 200 kHz, while turn-off loss rises from 42 μJ to 310 μJ over the same range (about a sevenfold increase).

Concurrently, peak module temperature increases steadily with frequency, following a nearly linear trend from 85 °C at 25 kHz to 109 °C at 200 kHz. Crucially, this maximum temperature of 109 °C remains well below the manufacturer-specified allowable junction temperature limit of 175 °C for the SiC devices—leaving a significant 66 °C thermal margin. This confirms that the module operates stably within the safe thermal design margin across the entire tested frequency spectrum, validating its reliability for high-frequency applications where thermal management is critical.

## 6. Conclusions

This paper proposes a closed-loop methodology of “design-simulation-process-validation”. Through in-depth synergy between electro-thermal simulations (LTspice/COMSOL) and micro/nano-packaging processes (sintering/bonding), a comprehensive closed-loop analysis of the SiC half-bridge power module is carried out regarding the “electro-thermal-process” coupling mechanism. The key findings and contributions are summarized as follows:

1. Dynamic switching characterization: A multifunctional double-pulse test board was designed to quantitatively analyze the dynamic switching characteristics of the SiC half-bridge power module under an 800 V operating condition. Experimental validation confirmed that the packaged module successfully withstood the 800 V high-voltage test, with the measured drain current (4.62 A) exhibiting a deviation of <0.65% from the simulated value (4.65 A). This demonstrates the accuracy of the electro-thermal co-design approach.
2. Thermal performance analysis: A COMSOL-based thermal simulation model was established to evaluate the temperature distribution of the module under a power loss of 117.6 W. The simulation revealed a junction temperature gradient of 55 °C, with localized hotspots concentrated at the SiC wafers. The maximum junction temperature (80 °C) remained significantly below the safety threshold (175 °C), validating the thermal reliability of the module.
3. Packaging process optimization: Key packaging parameters were quantified for reliability enhancement, including conductive adhesive sintering (S820-F680, thermal conductivity: 39.3 W/(m·K)), high-temperature baking at 150 °C, aluminum wire bonding (15-mil wire diameter, ultrasonic power = 500 mW, and bonding force = 500 g), and microscopic examination (Leica IVesta 3, 55× magnification) which confirmed defect-free sintering and bonding interfaces.
4. Frequency-dependent dynamic characteristics: Extraction of parasitic parameters for the power module was performed using ANSYS Q3D software, yielding a simulated inductance of 6 nH. Experimental validation via double-pulse testing measured the parasitic inductance at 8.3 nH. Both simulation and experimental results confirm the module’s relatively low parasitic parameters. Further evaluations across switching frequencies of 25 kHz to 200 kHz demonstrated a maximum temperature rise of 109 °C. This value remains below the 175 °C maximum junction temperature specified in the device datasheet, confirming operation within the permissible temperature range while maintaining low switching losses.

This study provides a process benchmark for the micro/nano-manufacturing of high-density SiC power modules, with direct application value especially in bonding process parameter optimization and interface reliability control [33,34].

However, the double-pulse test, while widely used for characterizing power semiconductor switching energies, faces limitations in loss measurement accuracy due to the following [26]:

1. Bandwidth constraints of probes, causing waveform distortion during fast SiC switching.
2. Timing mismatches between voltage/current probes, inducing phase errors.
3. Parasitic capacitances and inductances introducing extraneous energy components.

To address these limitations, future studies may adopt complementary methods, such as the thermal approach proposed in [26], which leverages junction temperature rise induced by self-heating effect to estimate switching energies.

Additionally, reference [35] compares the effects of different assembly methods (including sintering with the use of silver micropowder, soldering, gluing, and soldering with a silver sheet pad) on thermal resistance. Subsequent studies within this framework will further examine these specific assembly techniques to quantify their individual impacts on thermal resistance of the SiC half-bridge power module.

## Figures and Tables

**Table 1:** Research status of representative SiC half-bridge power modules.

Research Institutions	Research Status
North Dakota State University	Established an electro-thermal model to extract the temperature distribution of SiC modules, but failed to integrate practical process data (e.g., high-temperature baking), making it impossible to verify the model’s adaptability to high-temperature environments.
Aalborg University	Predicted SiC aging behavior using an electro-thermal co-simulation method, yet did not quantify the influence of sintering temperature/bonding parameters on the thermal model.
Huazhong University of Science and Technology	Explored an electro-thermal co-simulation method based on Pspice-MATLAB-COMSOL; however, it lacked practical process analysis experiments, making it difficult to evaluate the model’s robustness against process fluctuations.
University of Electronic Science and Technology of China	Investigated the design and experiments of packaging processes (e.g., substrate, wire bonding) for SiC power modules under high-temperature and high-voltage conditions, but did not perform thermal simulation analysis on module design, failing to predict the thermal distribution of power modules.
Virginia Polytechnic Institute and State University	In terms of packaging process design, it adopted lead-free bonding to minimize parasitic inductance. However, due to the lack of electrical simulation analysis on module design it was unable to predict its dynamic switching characteristics.

**Table 2:** LTspice simulation parameters for the double-pulse test circuit of the SiC half-bridge power module.

Parameters	Specifications
Test voltage $V_{CC}$	800 V
Simulation time T	17 μs
Load inductance L	850 μH
MOS for upper and lower bridge arms	HX1M013120W

**Table 3:** Characterization and impact of intra-module parasitic inductance.

Parameters	Definition	Specifications (nH)	Impact
L1, L4	Power loop inductance (drain path)	1.8, 1.6	Causing drain–source voltage oscillation or overshoot
L3, L6	Power loop inductance (source path)	1.2, 1.4	Causing drain–source voltage oscillation or overshoot
L2, L5	Gate–driver loop inductance	2.1, 1.9	Inducing gate-voltage oscillation

**Table 4:** The main material structure dimensions and positions.

Structure	Length (mm)	Width (mm)	Height (mm)	Coordinates (mm)
Upper-arm SiC MOS wafer	6.48	5.01	0.19	(x = 10, y = 11)
Lower-arm SiC MOS wafer	6.48	5.01	0.19	(x = 35, y = 11)
Copper substrate	45	22	1	(x = 22.5, y = 11)

**Table 5:** The information on the model of MOSFETs used in computations.

Material	Thermal ConductivityW/(m·K)	Density(kg/m^3^)	Heat Capacity at Constant Pressure [J/(kg·K)]
Cu	400	8940	385
SiC	450	3210	700
Alumina	27	3900	900

**Table 6:** The list of parameters values of the used model.

Parameters	Specifications
MOSFET wafer	HX1M013120W
Drain–source voltage	800 V
Continuous maximum drain current	10 A
On-resistance ($T_{j}=25\;{}^{\circ}C$)	0.45Ω
Threshold voltage ($V_{gs(th)}$)	15–25 V
Output capacitance ($C_{OSS}$)	70 pF
Operating junction and storage temperature range	−55~+175 °C

**Table 7:** The key parameters required for power loss calculation.

Parameters	Specifications
RMS (root mean square) current ($I_{d\text{\_}\mathrm{rms}}$)	100 A
Drain on-resistance ($R_{\mathrm{ds}}$)	23 mΩ
Conduction duty cycle (D)	0.5
Turn-on loss and turn-off loss ($E_{on}+E_{off}$)	105 μJ
Switching frequency ($f_{\mathrm{sw}}$)	25 kHz

**Table 8:** Main parameters and equipment models of the experimental platform.

Parameters	Specifications
Test voltage	800 V
Load inductance	850 uH
Gate-drive voltage of upper bridge	−6 V
Gate-drive voltage of lower bridge	21 V
Half-bridge SiC power module	HX1M013120W (Macrocore Semiconductor (Shenzhen, China))
High-voltage DC power supply	JingRi DP1200 (JingRi Technology Co., Ltd. (Hangzhou, China))
Low-voltage DC power supply	Siglent SPD3303C (Siglent Technologies Co., Ltd. (Shenzhen, China))
Oscilloscope	Siglent SDS2074X (Siglent Technologies Co., Ltd. (Shenzhen, China))
High-voltage differential probe	Siglent DPB5150A (Siglent Technologies Co., Ltd. (Shenzhen, China))
Rogowski coil	Micsig RCP600XS (Micsig Technologies Co., Ltd. (Shenzhen, China))

**Table 9:** Frequency-dependent switching characterization of power module.

Frequency (kHz)	Turn-On Loss (μJ)	Turn-Off Loss (μJ)	Peak Temperature (°C)
25	65	42	85
100	261	165	93
150	370	245	102
200	450	310	109

## Data Availability

Additional data are available upon request by contacting the corresponding author of this manuscript.
